# Data-driven comparison of multiple high-dimensional single-cell expression profiles

**DOI:** 10.1038/s10038-021-00989-9

**Published:** 2021-11-01

**Authors:** Daigo Okada, Jian Hao Cheng, Cheng Zheng, Ryo Yamada

**Affiliations:** Center for Genomic Medicine, Graduate School of Medicine, Kyoto University, Nanbusogo-Kenkyu-To-1, 5F, 53 Syogoin-Kawaramachi, Sakyo-ku, Kyoto, 606-8507 Japan

**Keywords:** Data mining, Gene expression

## Abstract

Comparing multiple single-cell expression datasets such as cytometry and scRNA-seq data between case and control donors provides information to elucidate the mechanisms of disease. We propose a completely data-driven computational biological method for this task. This overcomes the challenges of conventional cellular subset-based comparisons and facilitates further analyses such as machine learning and gene set analysis of single-cell expression datasets.

## Introduction

**Fig. 1:**
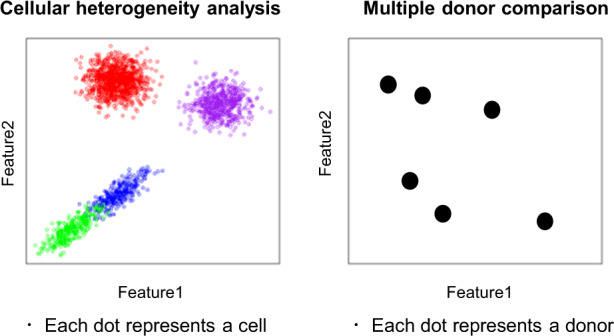
Conceptual difference between a cellular heterogeneity analysis and multi-donor comparison

Single-cell expression data, such as from cytometry and single-cell RNA-seq (scRNA-seq), provide information on cell population profiles. The greatest benefit of single-cell expression data is that it can reveal the heterogeneity of cell populations. Since scRNA-seq datasets contain many cells and many genes, computational methods to conduct dimensionality reduction in a data-driven manner such as PCA, tSNE, or UMAP are indispensable for their data analysis [1]. In such cellular heterogeneity analyses, the cells are embedded into a low-dimensional coordinate space (left panel in Fig. 1).

On the other hand, comparing cell population profiles between case and control donors provides information on the mechanism of complex diseases. Comparison of single-cell expression datasets from multiple donors requires data mining of datasets with donors, cells, and genes. This type of analysis is thus more complex than donor comparisons for bulk expression datasets with only donors and genes. When the purpose is comparing multiple donor profiles, the donors should be embedded into a low-dimensional coordinate space to be compared to each other (right panel in Fig. 1).

**Fig. 2:**
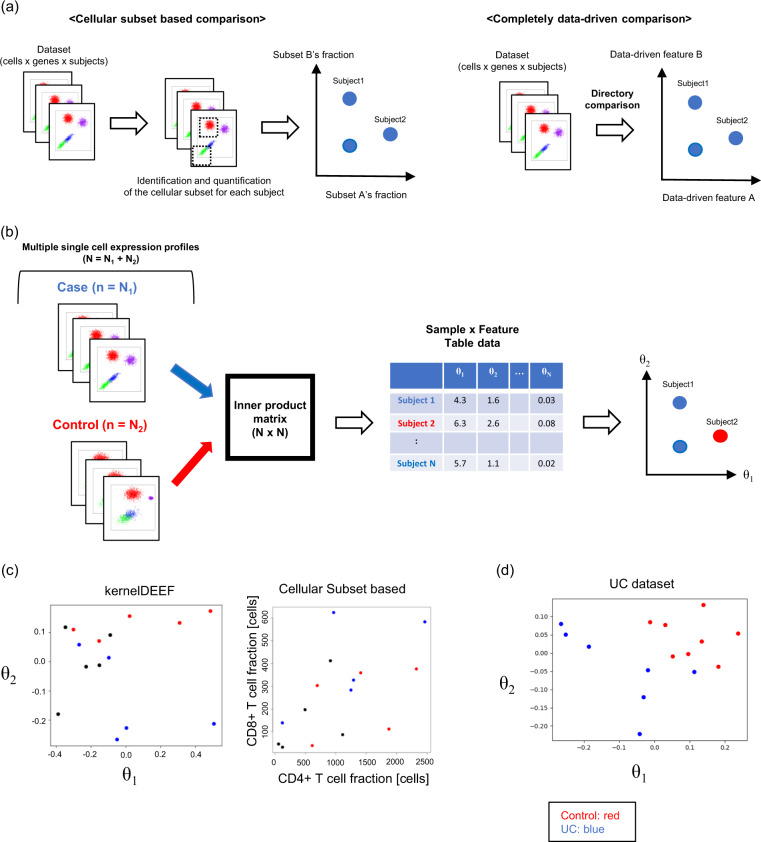
kernelDEEF method and its implementation. **a** Graphical explanation of conceptual differences between cellular subset-based and completely data-driven comparisons of multiple single-cell expression profiles. The completely data-driven method does not require biological subset annotation or multi-step processing like the cellular subset-based comparison, which allows for a direct comparison of the profiles among donors. **b** Graphical outline of the kernelDEEF algorithm for case/control analysis. **c** The result of feature extraction of kernelDEEF and conventional cellular subset-based methods for the ITN dataset. The left panel shows the kernelDEEF top two *θ* coordinates obtained in a completely data-driven manner. The right panel shows the number of CD4+ T cell and CD8+ T cell fractions in the profiles obtained by a conventional automatic gating approach. In kernelDEEF results, dissimilarities in PBMC profiles among patient groups described in the flowStats package are visualized. **d** The top two *θ* coordinate plots of the UC scRNA-seq dataset. The differences between UC and control donors are visualized on the plot

Analyses that compare multiple single-cell expression profiles have been performed mainly on cytometry data. The most common method is to construct a donor-by-cellular-subset matrix where the cellular subset fraction is identified and quantified using manual gating or computational methods. Once the datasets are converted to a donor-by-feature matrix, various computational biology methods can be applied. Alternatively, completely data-driven approaches that do not use any biological assumptions have been proposed for constructing a donor-by-feature matrix. Previously proposed approaches regarded single-cell expression data as just statistical samples from a probability distribution [2–4]. The merit of these types of methods is that they require no prior knowledge or assumptions, such as biological annotation of cellular subsets, and provide consistently data-driven workflow from donor, cell, and gene data to a donor-by-feature matrix. The differences between cellular subset-based and completely data-driven approaches are described in Fig. 2(a).

Recently, comparisons of multiple scRNA-seq datasets have also been discussed [5, 6]. Given that scRNA-seq contains much more information that is more complex than cytometry data, a completely data-driven approach would be very effective. However, existing completely data-driven methods are designed for cytometry data with few markers and cannot be applied to high-dimensional scRNA-seq, while computational biology methods for scRNA-seq data have mainly focused on cellular clustering or the identification of unknown subsets for one profile rather than for multiple profile comparisons. If a completely data-driven approach becomes possible for scRNA-seq, the various computational biology methods for multiple bulk RNA-seq datasets can be easily extended to scRNA-seq, such as machine learning or differential gene set analysis.

In this study, we propose a completely data-driven computational biological method for this task, which overcomes the challenges of conventional cellular subset-based comparisons. We also show two examples of applying this method: machine learning prediction of clinical cytometry data and differential distributed gene analysis for multiple scRNA-seq data.

## Results and discussion

### Completely data-driven comparisons of multiple single-cell expression profiles

We have developed a completely data-driven method, named kernelDEEF, to convert multiple high-dimensional single-cell expression profiles into a donor-by-feature matrix format in a completely data-driven manner (Fig. 2(b), detail in “Methods” section 1). First, it picks n cells from each donor and calculates the inner-product matrix between donors using a kernel method. Next, a matrix decomposition algorithm is applied to this inner-product matrix to give the coordinates of the donors based on the inner-product relationship. As with principal component analysis, these coordinates are calculated with the eigenvalues representing their contributions. We name the coordinates as *θ*_1_, *θ*_2_ ⋯ in the order of their contribution. Then, the top *θ* coordinates such as *θ*_1_ or *θ*_2_ are the data-driven features assigned to each donor. The advantage of kernelDEEF is that it can convert high-dimensional single-cell expression datasets (not only cytometry but also scRNA-seq) into a matrix format in a completely data-driven manner without any biological assumptions.

 Figure 2(c) shows an example of a multiple donors comparison with kernelDEEF (left panel) and cellular subsets (right panel) from a public cytometry dataset, ITN, which is a built-in dataset of the R package flowStats (see details in “Methods” sections 2-4) [7, 8]. The dataset consists of cytometry data of peripheral blood mononuclear cells (PBMCs) measured at five markers from 15 patients with patient group labels as described in the flowStats package. The left panel is *θ*_1_ and *θ*_2_ of kernelDEEF (n = 1000), and the right panel is the CD4+ T cell and CD8+ T cell fractions. In a cellular subset-based comparison, a multi-step gating process is applied, and only a portion of the information in the cellular population profile is used. kernelDEEF provides a consistent workflow for feature extraction, and the differences between patient groups are identified on *θ* coordinate plots.

We investigated the stability of our completely data-driven approach. We performed another two replications by cell resampling (n = 1000) and drew a coplot of *θ*_1_ and *θ*_2_ coordinate values, which suggested that the result is quite stable for the resampling (Additional File 1). Using this resampling analysis, it can be estimated whether the results of kernelDEEF are affected by sampling bias. Additional File 2 is the result in a case when the inner-product was calculated not by the kernel function but by a grid-wise probability estimation. It shows a similar pattern as the *θ* coordinate plot (see details in “Methods” section 4).

Figure 2(d) shows the *θ* coordinate plots of scRNA-seq data of PBMCs from patients with ulcerative colitis (UC), a representative inflammatory bowel disease, and control donors [9]. After preprocessing, 876 genes were included in the analysis (see details of the dataset and preprocessing in “Methods” section 4). The number of cells chosen (n) was set to 672, because this was the minimum number of cells that could be included in the profiles. The differences between UC and control donors are clearly visualized on the top two *θ* coordinates. The control donors were observed in a region where both *θ*_1_ and *θ*_2_ values are large. Although the sample size was quite small, two groups of UC donors were observed: four donors with small *θ*_2_, and three donors with large *θ*_2_. If a larger sample size becomes available in a scRNA-seq study, it may be possible to classify disease subtypes based on their cell population expression profiles.

Another possible application of this method is the detection of batch effects in single-cell expression datasets. If it is possible that there are batch effects in the dataset, we can visualize the effects by changing the disease/control label to batch. In addition, the effects can be tested and corrected in the same way as for ordinary multivariate data. In fact, this can be done for the batch effect in large-scale cytometry data using a conventional completely data-driven approach [10]. While this approach is only applicable to low-dimensional data, kernelDEEF is also capable of fully data-driven batch effect removal for high-dimensional single-cell expression data.

Our method was able to convert multiple single-cell expression datasets into a donor-by-*θ* coordinate matrix in a completely data-driven manner to compare donors. After this procedure, we can apply various unsupervised learning methods in data science field to this matrix. In addition, when the donors have disease/control labels or other measurements, supervised machine learning or association analyses can be performed. In the following sections, we propose two applications of this *θ* coordinate matrix: machine learning prediction of clinical cytometry data and differential distributed gene analysis for multiple scRNA-seq data.

### Supervised machine learning for multiple clinical cytometry datasets

**Fig. 3:**
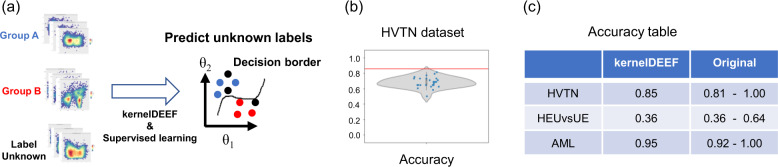
The results of supervised machine learning for multiple clinical cytometry datasets. **a** Graphical outline of machine learning prediction task of the cytometry data. **b** Result of machine learning of the HVTN dataset. The violin plot of the accuracy for 25 resamplings with that of the ensemble prediction with all resamplings is shown (red line). **c** Table of prediction accuracy. The first column shows the prediction accuracy of our approach on the test data for the three datasets. The second column shows the maximum and minimum accuracy of the competition as described in the original paper

We developed a kernelDEEF-based supervised machine learning workflow to predict the donor label from cytometry data (Fig. 3(a)). Cytometry is also used in clinical practice, and the development of machine learning technology to diagnose diseases from cytometry data is an important topic. In this workflow, we applied a supervised machine learning algorithm, XGBoost, to a donor-by-*θ* coordinate matrix. We employed resampling and an ensemble to improve the performance of the prediction, where the training and prediction were performed on different multiple statistical samples by cellular resampling, and the final predictive label was determined by a majority vote of these predictions.

We applied this workflow to three datasets (HVTN, AML, and HEU vs. UE) that were used in a previous competition for label prediction of cytometry data [11]. This previous paper reported the machine learning results of many computational cytometry methods for cellular subset identification. Dataset HVTN is a T cell cytometry dataset consisting of 96 samples stimulated with either GAG antigen or ETV antigen. HEU vs. UE and the AML dataset are different data structures where cytometry data under multiple conditions are measured for each donor. HEU vs. UE has 44 donors, either HEU (exposed to HIV in utero but uninfected) or UE (unexposed). For each donor, seven conditions (stimulated by six different drugs (CPG, LPS, PAM, PG, PIC, and R848) and unstimulated) of T cell cytometry data were measured. The AML dataset has 380 donors, either acute myeloid leukemia (AML) or normal. For each donor, eight conditions (tube1-tube8) of T cell cytometry data were measured. In HEU vs. UE and AML, machine learning was performed on the integrated matrix created by concatenating *θ* coordinate matrices of all conditions in the columns. In all cases, we split the data into training and test data under the same conditions as in the original paper and examined the prediction accuracy of our workflow. Details of the workflow are described in “Methods” section 6.

Figure 3(b) shows the result of the HVTN dataset. The ensemble of 25 resamplings showed higher accuracy than all 25 resamplings (accuracy = 0.85). This result suggests that our resampling and ensemble procedure is effective for improving accuracy. Fig. 3(c) shows the accuracy of all three datasets and the accuracy of the original study results. HVTN and AML do not show higher accuracy but stable performance. HEU vs. UE shows poor performance as in the original study, though it was concluded in the original paper that this label classification was a difficult task. Violin plots of the accuracies of HEU vs. UE and AML are shown in Additional File 3 and Additional File 4, respectively.

These results suggest that the kernelDEEF-based machine learning workflow has stable performance in a completely data-driven manner. The advantage of this method is that it allows a data scientist to perform machine learning prediction on arbitrary cytometry datasets without any biological knowledge. However, the accuracy of our method was not higher than that of the conventional approach. The reason is likely that a larger sample size is needed to achieve higher performance, since kernelDEEF uses nonparametric methods.

### Differential distributed gene analysis for multiple scRNA-seq datasets

**Fig. 4:**
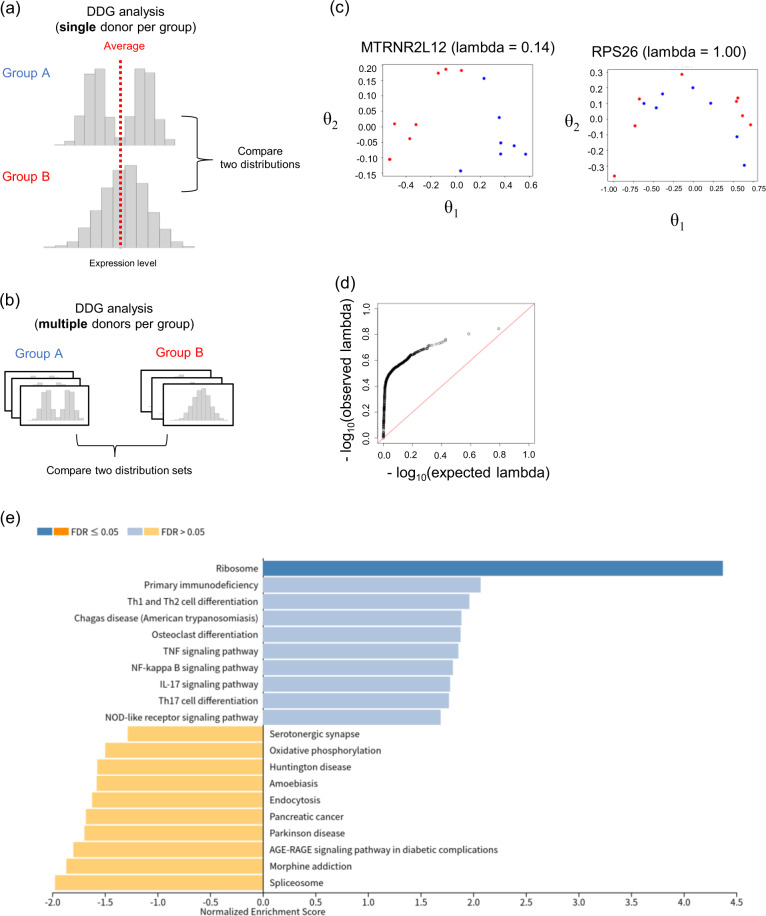
The results of DDG analysis for multiple scRNA-seq datasets. **a** Graphical illustration of standard DDG analysis. It compares the two distributions of expression levels of single cells and identifies the genes differentially distributed between groups such as case or control. Even if the average expression level is same, the difference in expression distribution can be detected. **b** Our setting of DDG analysis in a situation with multiple donors per label using kernelDEEF. **c** Plot of the top two *θ* coordinates for the genes with the smallest (left panel) and largest (right panel) Wilks' lambda in the DDG analysis of the UC scRNA-seq dataset. Red points: control donors. Blue points: UC donors. Note that the two red points are plotted very close to each other and appear to overlap in the left panel. **d** QQ plot to compare the distribution of lambda (y-axis) with the null distribution obtained from the label permutation (x-axis) where the red line represents y = x. **e** KEGG pathways listed by GSEA for Wilks' lambda. The figure is a bar chart of the default output of WebGestalt, showing the top 10 relatively stable pathways (blue) and differential pathways (yellow)

One of the merits of single-cell RNA-seq is that it can identify differential distributed genes (DDG) between two groups [12]. DDG analysis identifies genes with differences in the distribution of their expression levels in single cells between groups (Fig. 4(a)). In the case of the bulk transcriptome, only the average expression levels in the cell population can be observed. Differential expressed gene (DEG) analysis using bulk transcriptome data compares these observations between groups and identifies the relevant genes. Therefore, DDG analysis using scRNA-seq has the potential to detect more diverse differences in gene expression patterns between groups even if the average expression level is same.

Although DDG analysis is a powerful approach that takes advantage of the characteristics of single-cell data, previous studies have used only one scRNA-seq profile per group, making it difficult to distinguish between individual differences and group differences. Using a *θ* coordinate matrix of kernelDEEF, it is possible to identify the DDG from multiple scRNA-seq datasets (Fig. 4(b)). DDG analysis for multiple donors per group would be a natural extension of DEG analysis in bulk datasets.

We developed a kernelDEEF-based DDG analysis designed for multiple profiles and applied it to the UC dataset (detailed in Methods section 7). First, the top two *θ* coordinates of donors were calculated by applying kernelDEEF to each gene expression distribution. Next, we obtained each gene’s Wilks’ lambda, which is a statistic of the multivariate analysis of variance and represents the label separation on the coordinate space. Genes with a small lambda are differentially distributed genes between disease and control. As an example, Fig. 4(c) shows plots of genes with the smallest and largest lambda in the UC dataset. Interestingly, the QQ plot of lambda values suggests that a majority of the genes were differentially distributed between labels (Fig. 4(d)), where the expected null distribution of the lambda was calculated by the permutation of the label once for each gene.

We conducted a validation through the resampling procedure and a comparison with the conventional method. First, three resamplings suggested that *λ* is stable for resampling, with the Spearman correlation coefficient of *λ* among different resamplings being > 0.8 (the coplot is shown in Additional File 5). We also applied the conventional DDG method, scDD, to this dataset (see details in Methods section 8), and returned a similar pattern of the QQ plot of the P-value distributions (Additional File 6), which also indicated that a majority of the genes were differentially distributed. A correlation was also observed between our *λ* values and scDD P values (Additional File 7).

Next, we applied Gene Set Enrichment Analysis (GSEA) for lambda values to investigate differential and relatively stable pathways of PBMC populations between UC and control. Fig. 4(e) shows the top ten KEGG pathways for relatively stable (blue) and differential (yellow) genes. The ribosome pathway was the most stable pathway. Interestingly, the top differential pathways were associated with neurodegenerative disorders such as morphine addiction, Parkinson’s disease, or Huntington’s disease rather than immunological pathways. A relationship between neurodegenerative diseases and inflammatory bowel disease has been previously noted [13], and it has been suggested that gut bacteria may be related to neurological diseases [14]. These analyses add to our understanding of the pathways involved in differences in cell population profiles.

## Conclusion

In this study, we proposed a completely data-driven computational method comparing multiple high-dimensional single-cell expression datasets with donors, cells, and genes. In this method, datasets are converted to a donor-by-feature matrix, which overcomes the challenges of conventional cellular subset-based conversions. By using our framework, most of the bulk data analysis methods can be extended to single-cell data with case/control labels in a natural way such as machine learning label prediction or gene set analysis. The results of this study suggest that preparing multiple donors for case/control studies may lead to new discoveries, even with single-cell expression study designs.

## Method

### kernelDEEF method

In this method, single-cell expression data can be thought of as a statistical sample from a high-dimensional probability distribution. The first step is to calculate the inner-product matrix between the donors. For the inner-product of the distributions, we use an index based on kernel mean embedding [15]. We picked n cells from each profile with no duplication allowed and calculated the inner-product of the donors. The index calculated as follows was used as the inner-product of the two probability distributions _*pi*_ and *p*_*j*_ from which the i-th and j-th profiles were sampled.

1 $$ < {p}_{i},{p}_{j} > =\frac{1}{{n}^{2}}\mathop{\sum }\limits_{k}^{n}\mathop{\sum }\limits_{l}^{n}K({{{{{{{{\bf{x}}}}}}}}}_{ik},{{{{{{{{\bf{x}}}}}}}}}_{jl})$$

where *x*_*ik*_, *x*_*jl*_ is the gene/marker expression vector of the k-th cell from the i-th profile and the gene/marker expression vector of the l-th cell from the j-th profile. *K*(*x*_*ik*_, *x*_*jl*_) is the kernel function, and a radial basis function kernel was adopted as follows:

2 $$K\left({{{{{{{{\bf{x}}}}}}}}}_{ik},{{{{{{{{\bf{x}}}}}}}}}_{jl}\right)=\exp \left(-\gamma {\left\Vert {{{{{{{{\bf{x}}}}}}}}}_{ik}-{{{{{{{{\bf{x}}}}}}}}}_{jl}\right\Vert }^{2}\right)$$

*γ* is the hyper-parameter, and we automatically set this value as $\frac{1}{p}$ [16]. Next, we gave each donor a coordinate based on this inner-product matrix. We adopted the DEEF method, which gives coordinates based on the inner-product of the probability distributions [4]. This method applies eigenvalue decomposition of the N by N matrix, whose (i, j)-th element is $\frac{1}{2}\;{{{{{{\mathrm{log}}}}}}}\, < {p}_{i},{p}_{j} >$. ***θ***_***i***_ is defined as the product of the i-th eigenvector and square root of the absolute i-th eigenvalue, and can be treated as a data-driven distribution feature. Therefore, [***θ***_***1***_, ***θ***_***2***_ ⋯ ***θ***_***N***_] is a matrix of a donor-by-*θ* coordinate values where the subscripts indicate the order of the contributions expressed in the eigenvalues from the largest positive (*θ*_1_) to the largest negative value. As a result, the information in the dataset is converted from a donor to a feature matrix.

### Cytometry dataset ITN

We used a publicly available cytometry dataset (ITN) as an example of cytometry implementation. The ITN dataset is a built-in cytometry dataset of the R package flowStats [7, 8]. The dataset contains PBMCs with five markers (CD8, CD69, CD4, CD3, and HLADr) from 15 patients in different patient groups as described in the flowStats package. Each profile in this dataset contains 10,000 cells.

### Automatic cellular subset quantification for the ITN dataset

We performed automatic cellular subset fraction quantification with the code described in the vignette document of the flowStats package. Here, CD4+ and CD8+ T cells were identified by sequential application of CD3/SSC lymphocyte gating and CD4/CD8 gating.

### A complete data-driven approach for the ITN dataset

All raw profiles in the ITN dataset were transformed by asinh(intensity/5) for downstream analysis as a complete data-driven approach.

We applied kernelDEEF to this dataset (*n* = 1000). For a comparison with kernelDEEF, we also performed a grid-wise inner-product calculation procedure using the ITN dataset [4]. Here, the range was set to include the range between *α* and 1 − *α* percentile for each marker (*α* = 0.15). The range of expression values for this marker was divided into a grid of 10. Since the number of markers is five, the total number of grids is 10^5^. For these grids, a density estimation was performed using the k nearest neighbor method (k = 100). The obtained densities were normalized to sum to 1, and the inner-products were calculated by vector calculation. The coordinates were obtained by applying DEEF to the inner-product matrix obtained in this way.

### scRNA-seq dataset UC and its preprocessing

We obtained the scRNA-seq data of patients with UC and control donors from NCBI GEO (GSE125527) [9]. We used the processed PBMC data of 15 donors (seven UC and eight healthy, GSM3576411-GSM357642). We performed quality control using Seurat under the following criteria: cells with nFeature_RNA < 200, nFeature_RNA > 7000, nCount_RNA > 70,000, or percent.mt > 10 were removed [17, 18]. Next, log normalization was applied to the dataset using the Seurat NormalizeData function where the scale.factor parameter was 10,000. We used genes passing the pooled sum of the expression value of all cells and all samples > 15,000 for the downstream analysis. The reasons for this gene filtering are as follows: first, a certain amount of expression is desirable for robust quantification of distributional dissimilarities per gene, and second, to reduce computational cost. After the filtering of genes, we finally used 876 genes.

### Machine learning classification of cytometry data

We used three datasets (HVTN, HEU vs. UE, and AML) from a previous study [11]. HVTN, HEU vs. UE, and AML were downloaded from the Flow Repository [19] (IDs: FR-FCM-ZZZV, FR-FCM-ZZZU, and FR-FCM-ZZYA, respectively). The HVTN dataset includes post-HIV vaccination T cell profiles after stimulation by two types of antigen, namely ENV and GAG. This dataset contains 96 samples (48 GAG samples and 48 ENV samples) in which the following protein markers were measured: CD4, TNFa, IL4 IFNg, CD8, CD3, and IL2. The HEU vs. UE dataset contains 308 samples from 44 donors (20 HEU and 24 UE). For each donor, cytometry measurements were taken under seven different conditions stimulated by six different drugs (CPG, LPS, PAM, PG, PIC, and R848) and unstimulated, and the following protein markers were measured: IFNa, CD123, MHCII, CD14, CD11c, IL6, IL12, and TNFa. The AML dataset contains 2872 samples from 359 donors (43 AML and 316 normal) in which the following protein markers were measured: IgG1-FITC, IgG1-PE, CD45-ECD, IgG1-PC5, and IgG1-PC7. For each donor, eight conditions (tube 1-tube 8) of T cell cytometry data were measured. For all datasets, asinh(intensity/5) was applied as a normalization preprocess.

We applied the kernelDEEF procedure and calculated donors by the *θ* coordinates matrix, where the number of picked cells (n) was 1000. Only the coordinate axes corresponding to positive eigenvalues were used in subsequent analyses. These matrix data were used for the subsequent machine learning procedure. In the HVTN data implementation, the training data and test data were described in the original dataset, then we used the same split (the proportion of training data was 50%). Because the training and test labels were not described in AML and HEU vs. UE datasets, the datasets were randomly split to have the same training rate (50%) as in the original paper.

We used the XGBoost algorithm for sample classification with the Python library xgboost, where n_estimators = 5000 [20]. The hyper-parameter max_depth (2, 3, 4, or 5) and the number of top *θ* coordinates to use were determined with the GridSerchCV function of scikit-learn, where the fold-number of cross-validation was set to five. To reduce the calculation burden of the grid search for how many top *θ* coordinates to use, the number of *θ* coordinates to be used was increased in order starting from 1, and if the best score of the cross-validation was not updated more than 11 times, the search was stopped. We conducted different cell resamplings and ensembles. After repeating the above prediction 25 times, the final predictive label was determined by a majority vote of these predictions. For AML and HEU vs. UE, after determining the optimal number of coordinates for each condition, XGBoost was applied to the integrated matrix created by concatenating *θ* coordinate matrices of all conditions in the columns.

### kernelDEEF-based differential distributed gene analysis procedure

We used the top two *θ* coordinates for the downstream analysis. For each gene, we calculated the Wilks’ lambda using the Python statsmodels library’s multivariate.manova.MANOVA function [21]. In addition, we computed the lambda under the permutation of the label once for each gene and created a null distribution for lambda. We applied GSEA for the lambda values to investigate which gene set tended to be differently distributed. GSEA was performed by WebGestalt (WEB-based GEne SeT AnaLysis Toolkit) with default settings for KEGG pathways [22].

### Comparison with scDD results

We sampled 672 cells from each donor and pooled them into UC and control groups, respectively. As such, the UC group contained 4704 (672 × 7) cells, and the control group contained 5376 (672 × 8) cells. scDD was applied using the R package to the expression data of these two groups with default settings[12].

## Supplementary information

Additional File 1. Coplot of coordinate values for θ1 and θ2 in three cell resamplings (Rep1, Rep2, Rep3) with the ITN dataset.

Additional File 2. Top two θ coordinate plots of the ITN dataset with grid-wise estimated inner products and DEEF applied.

Additional File 3. Performance in the HEU vs. UE dataset.

Additional File 4. Performance in the AML dataset.

Additional File 5. Coplot of Wiks’ lambda values of the genes for the three resamplings (Rep1, Rep2, Rep3) in the UC dataset.

Additional File 6. QQ plot to compare the distribution of -log10(P value) calculated from scDD (observed, y axis) with the null distribution obtained from the label permutation (expected, x axis).

Additional File 7. Coplot to compare -log10(lambda) calculated from our method (x axis) and -log10(P value) calculated from scDD (y axis).

## Data Availability

We created a brief tutorial for kernelDEEF using Python with the Jupyter notebook at https://github.com/DaigoOkada/kernel_deef_tutorial. The code used in this study is available at https://github.com/DaigoOkada/kernel_deef_code.
