# Supplementary figures and images for: Data-driven comparison of multiple high-dimensional single-cell expression profiles
Source: J Hum Genet. 2021 Nov 1;67(4):215–21. doi: 10.1038/s10038-021-00989-9 (PMC8948086; doi:10.1038/s10038-021-00989-9)

ITN dataset,  $\theta_1$

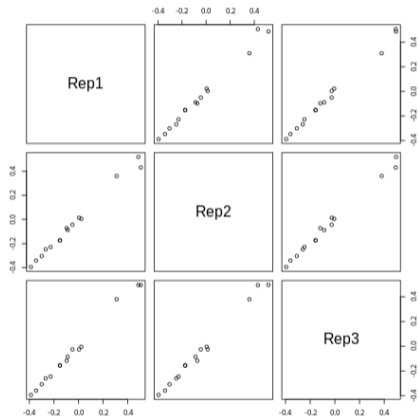

ITN dataset,  $\theta_2$

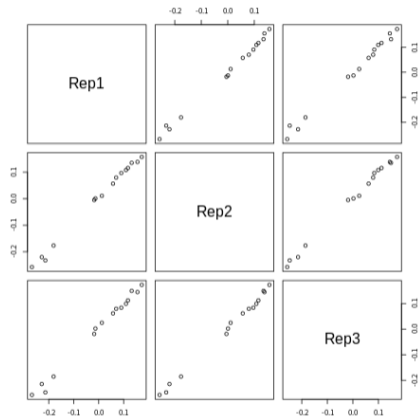

Supplement: Supplementary file 1 — Additional File 1. Coplot of coordinate values for θ1 and θ2 in three cell resamplings (Rep1, Rep2, Rep3) with the ITN dataset. [file 10038_2021_989_MOESM1_ESM.pdf]

DEEF

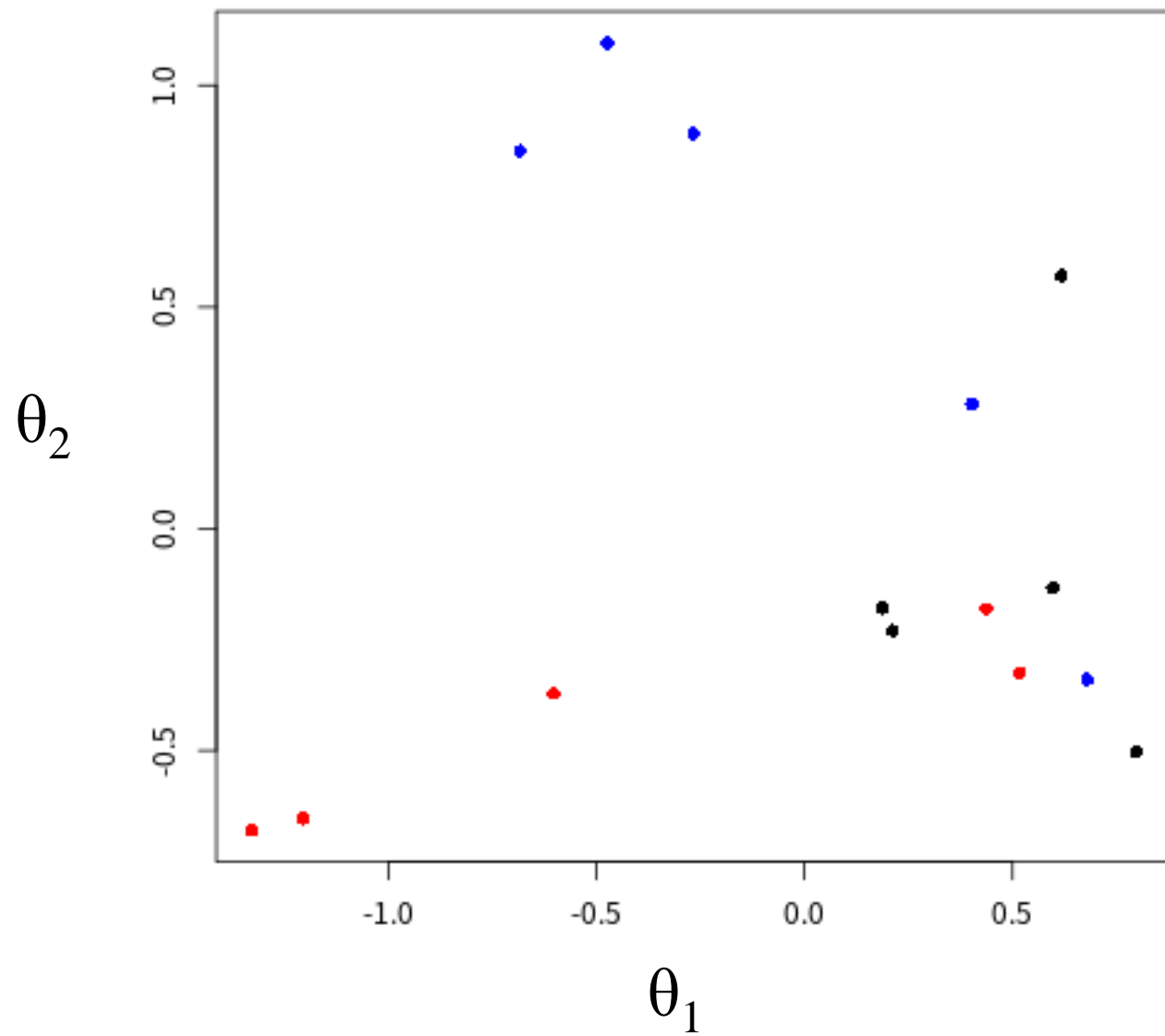

Supplement: Supplementary file 2 — Additional File 2. Top two θ coordinate plots of the ITN dataset with grid-wise estimated inner products and DEEF applied. [file 10038_2021_989_MOESM2_ESM.pdf]

# Integrated

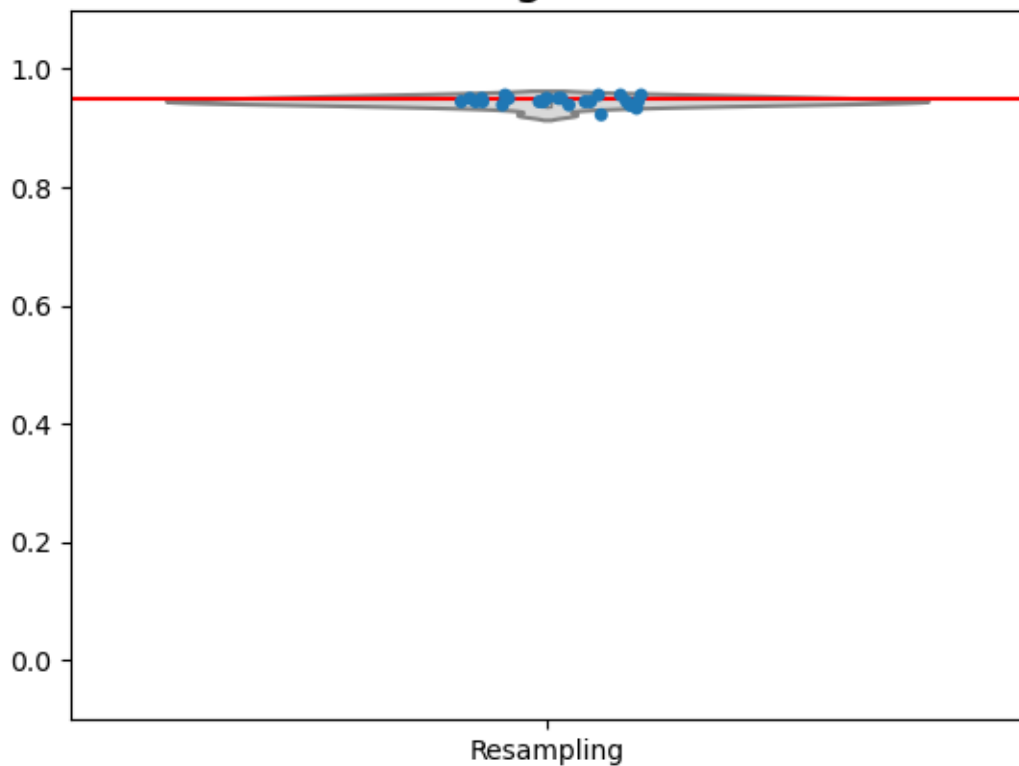

# Tube1

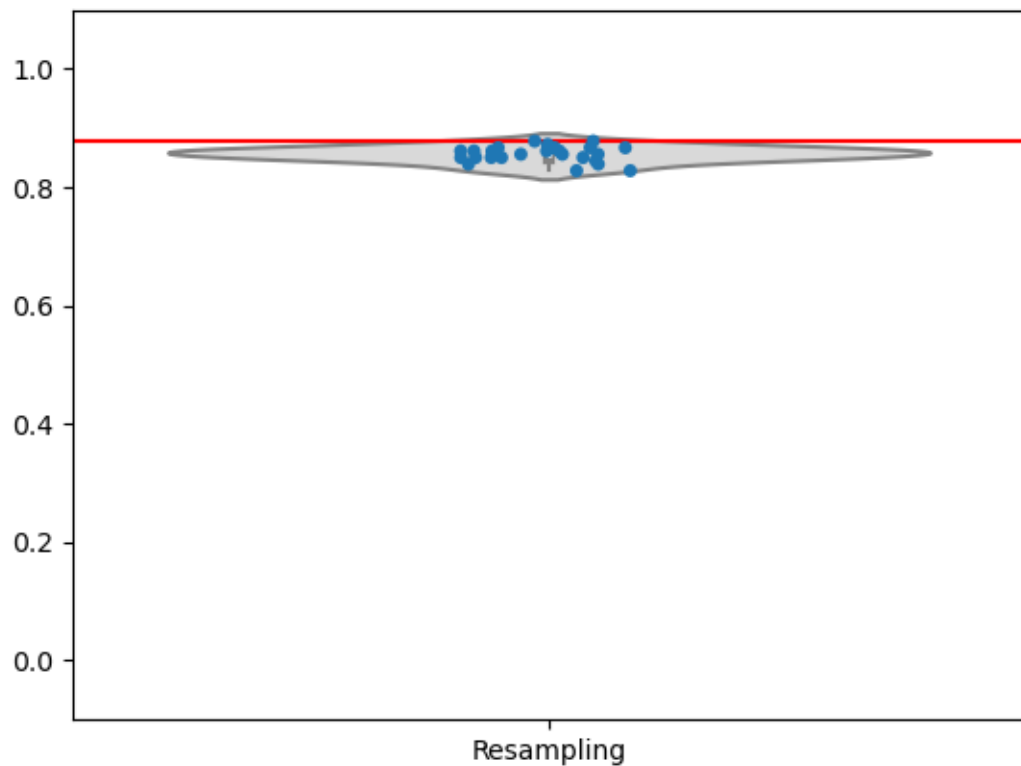

## Tube2

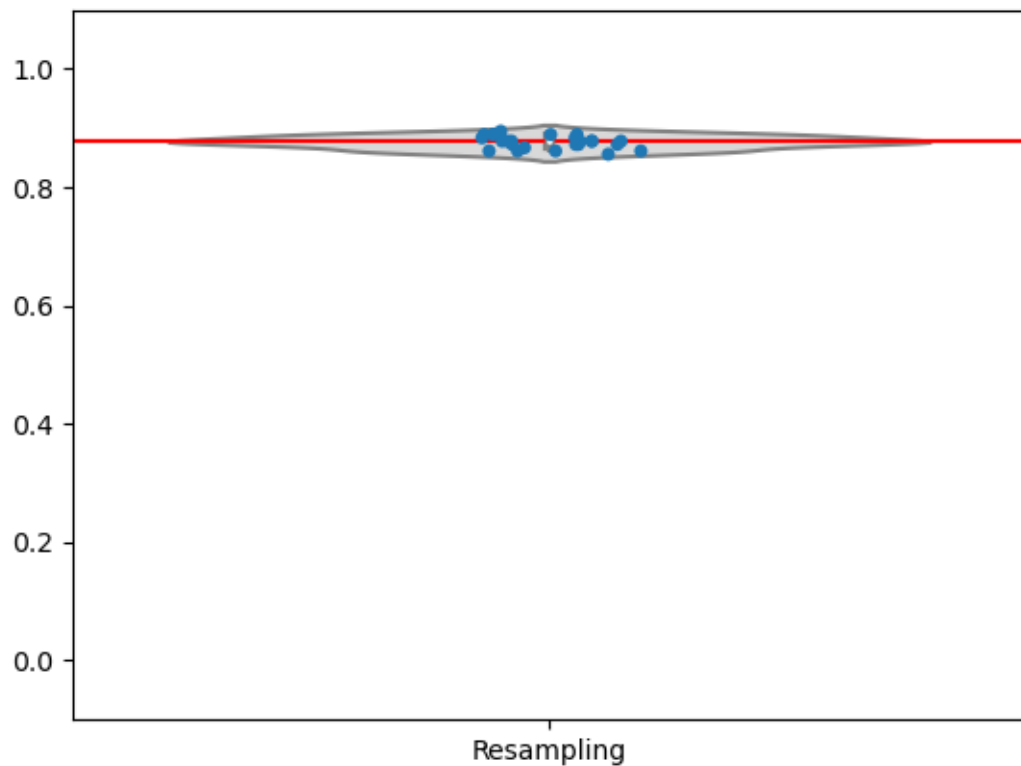

# Tube3

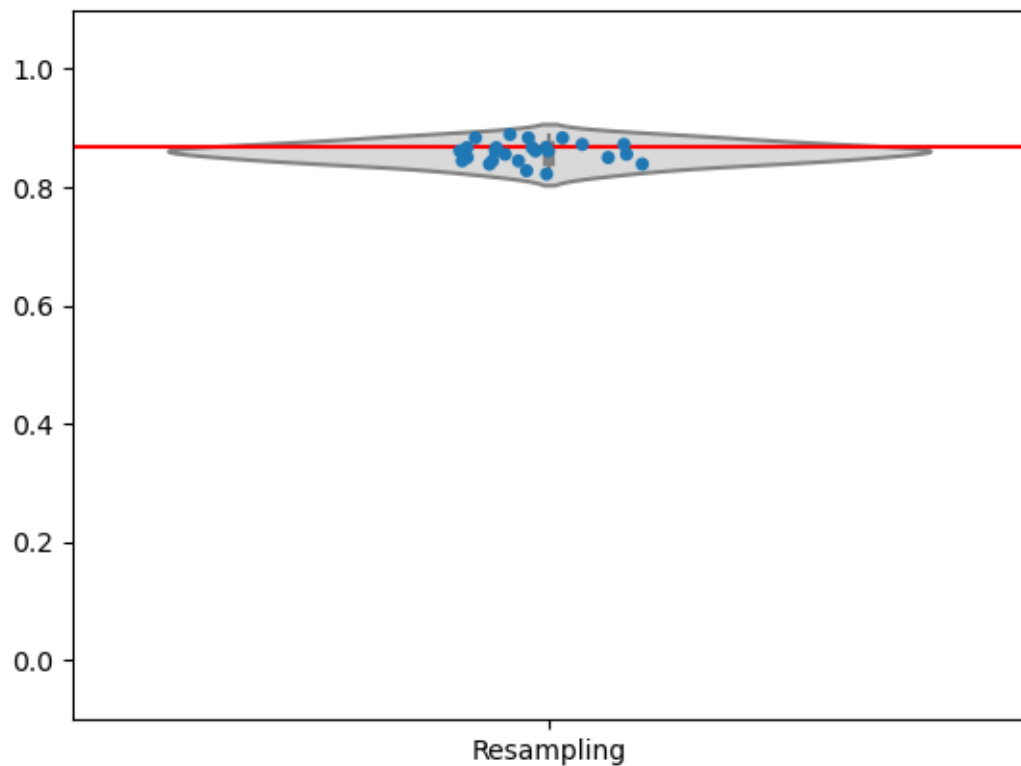

# Tube4

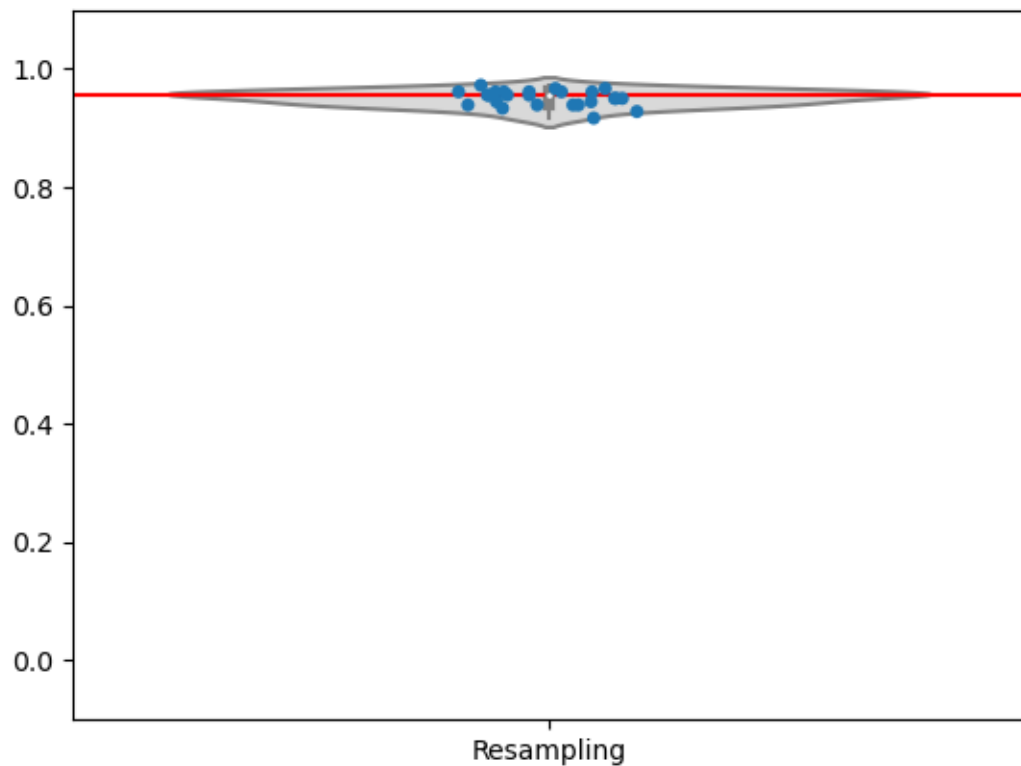

# Tube5

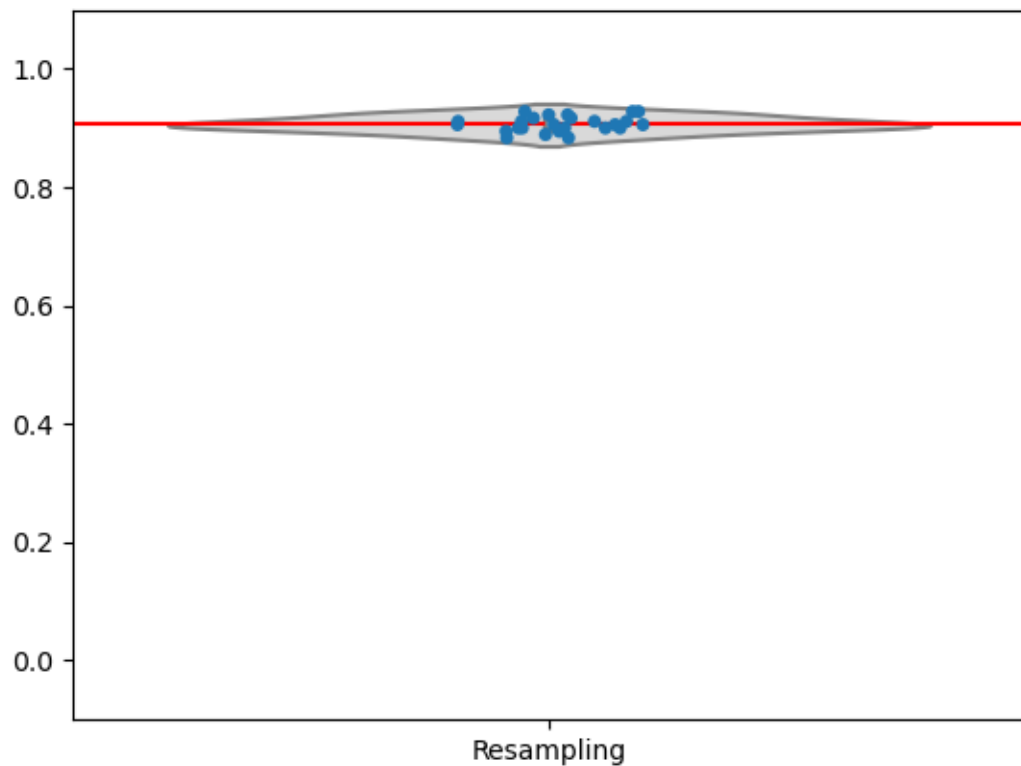

# Tube6

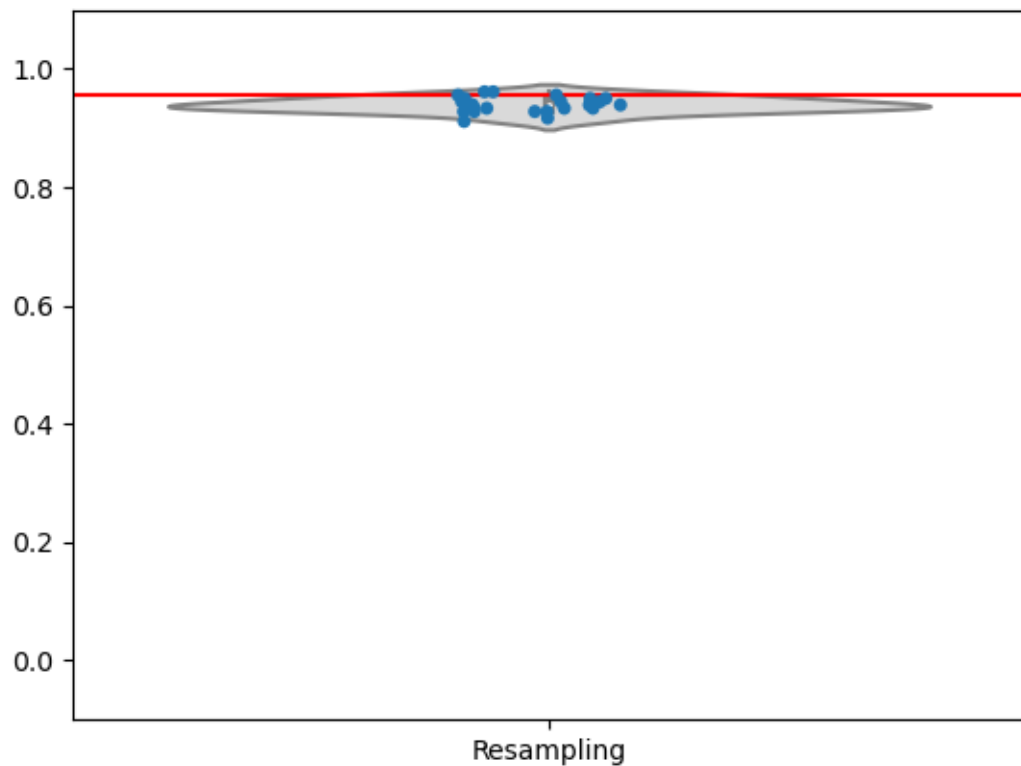

# Tube7

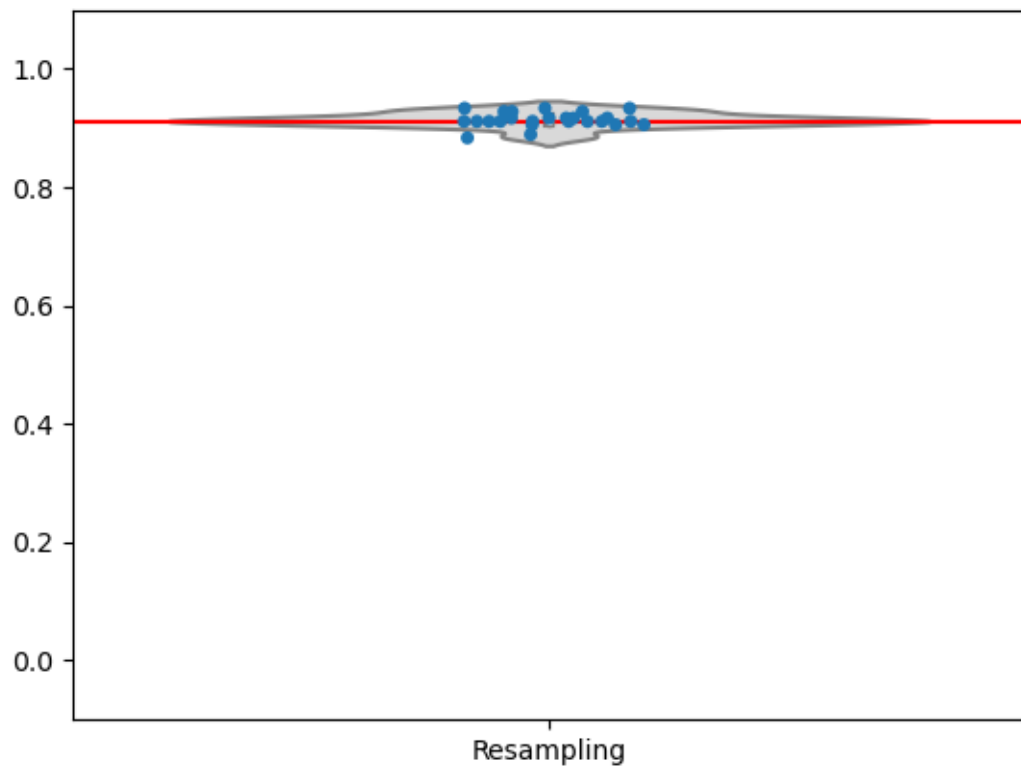

# Tube8

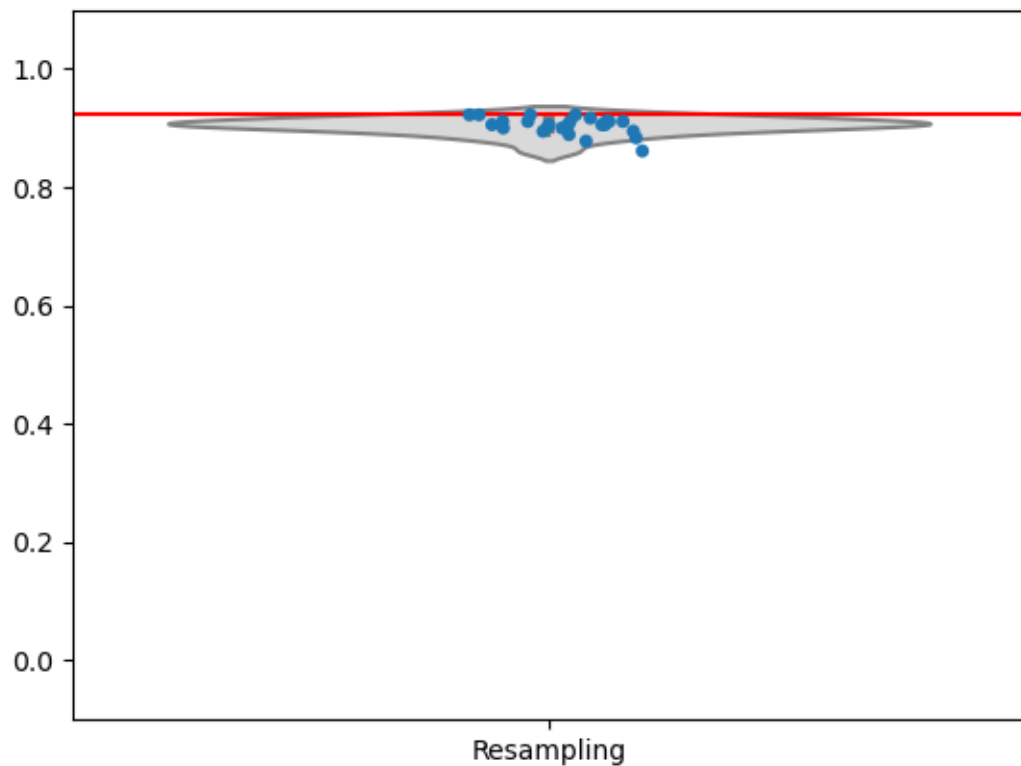

Supplement: Supplementary file 4 — Additional File 4. Performance in the AML dataset. [file 10038_2021_989_MOESM4_ESM.pdf]

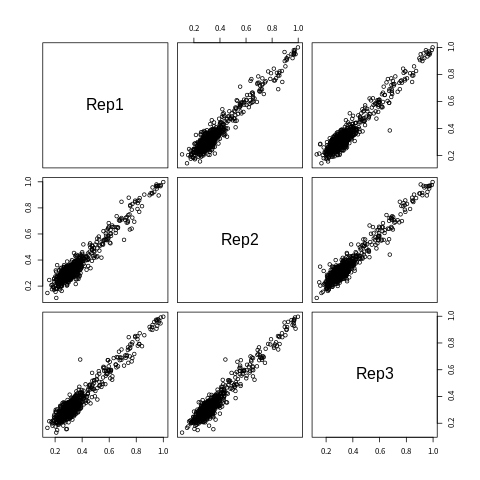

Supplement: Supplementary file 5 — Additional File 5. Coplot of Wiks’ lambda values of the genes for the three resamplings (Rep1, Rep2, Rep3) in the UC dataset. [file 10038_2021_989_MOESM5_ESM.png]

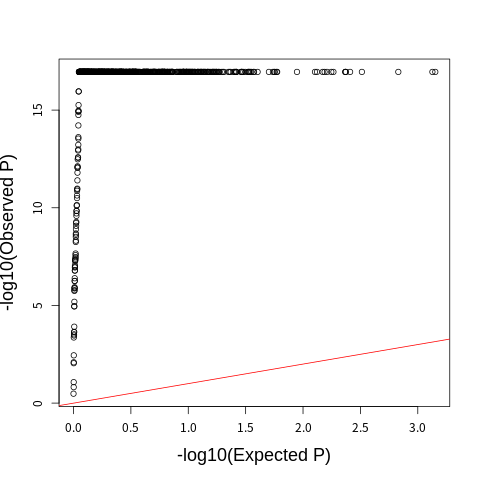

Supplement: Supplementary file 6 — Additional File 6. QQ plot to compare the distribution of -log10(P value) calculated from scDD (observed, y axis) with the null distribution obtained from the label permutation (expected, x axis). [file 10038_2021_989_MOESM6_ESM.png]

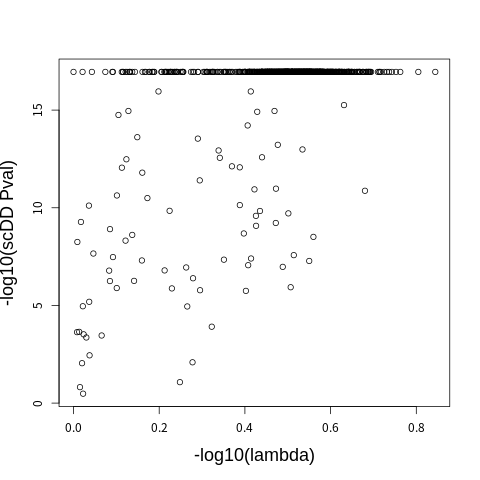

Supplement: Supplementary file 7 — Additional File 7. Coplot to compare -log10(lambda) calculated from our method (x axis) and -log10(P value) calculated from scDD (y axis). [file 10038_2021_989_MOESM7_ESM.png]
