# Supplementary material for: Data-driven comparison of multiple high-dimensional single-cell expression profiles
Source: J Hum Genet. 2021 Nov 1;67(4):215–21. doi: 10.1038/s10038-021-00989-9 (PMC8948086; doi:10.1038/s10038-021-00989-9)
Supplement: Supplementary file 3 — Additional File 3. Performance in the HEU vs. UE dataset. [file 10038_2021_989_MOESM3_ESM.pdf]

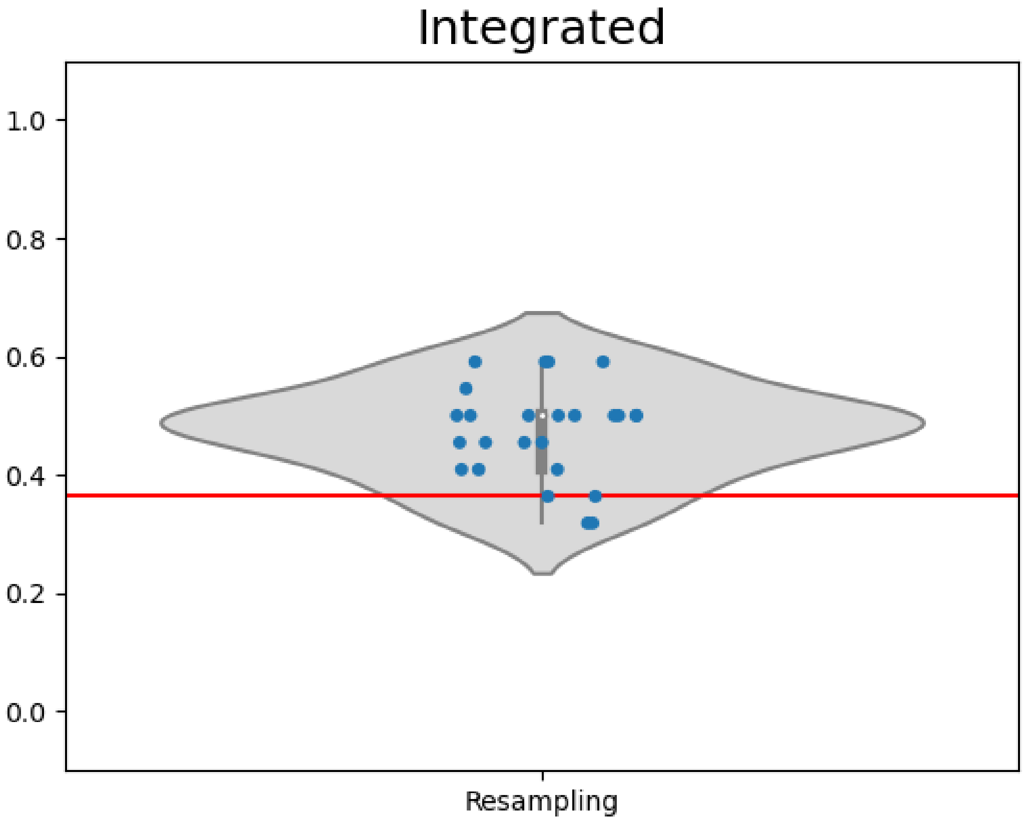
Integrated
1.0
0.8
0.6
0.4
0.2
0.0
Resampling

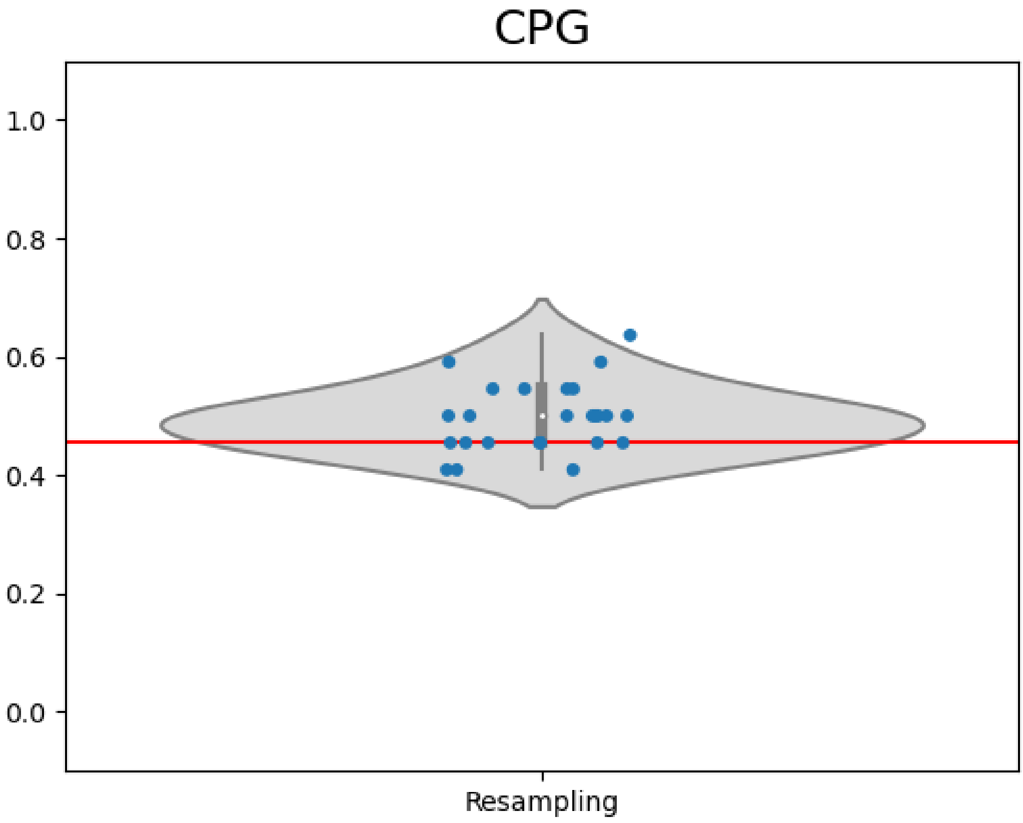
CPG
1.0
0.8
0.6
0.4
0.2
0.0
Resampling

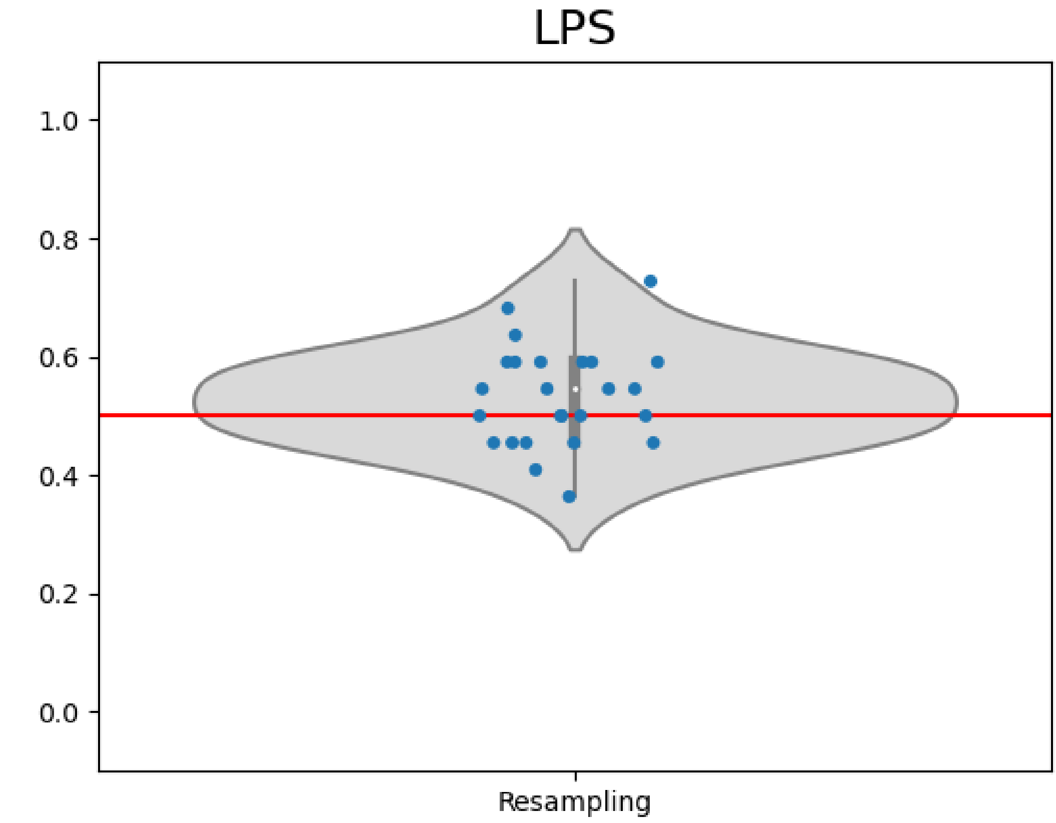
LPS
1.0
0.8
0.6
0.4
0.2
0.0
Resampling

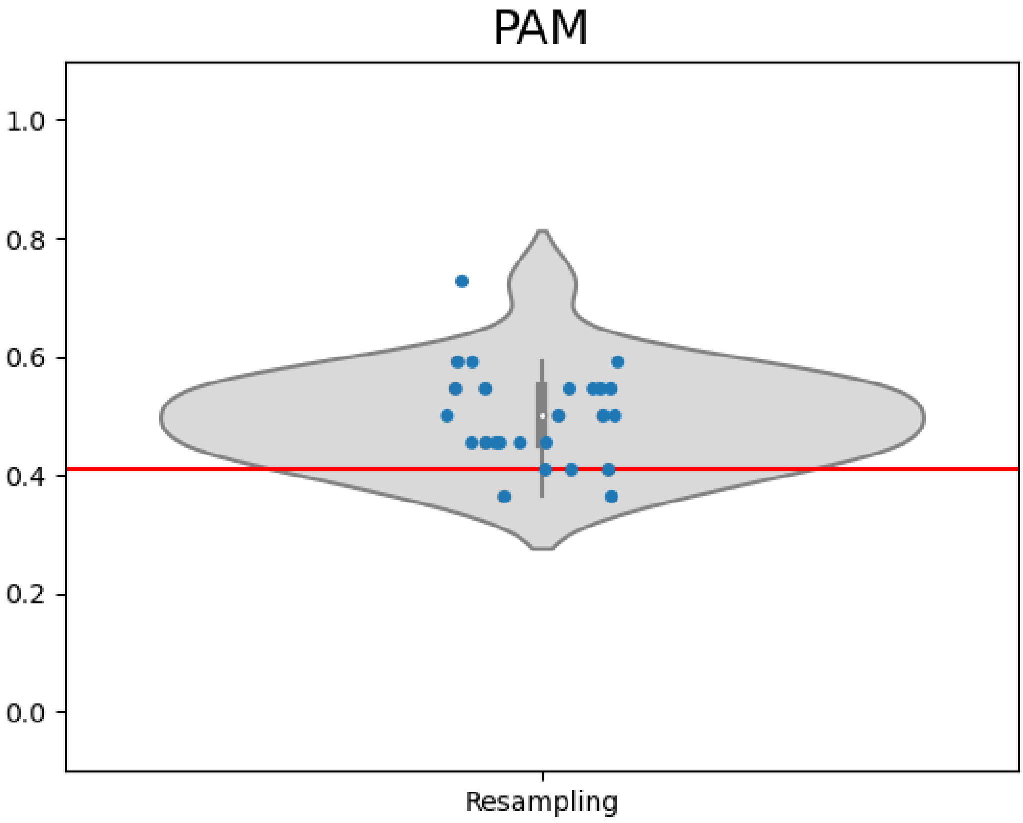
PAM
1.0
0.8
0.6
0.4
0.2
0.0
Resampling

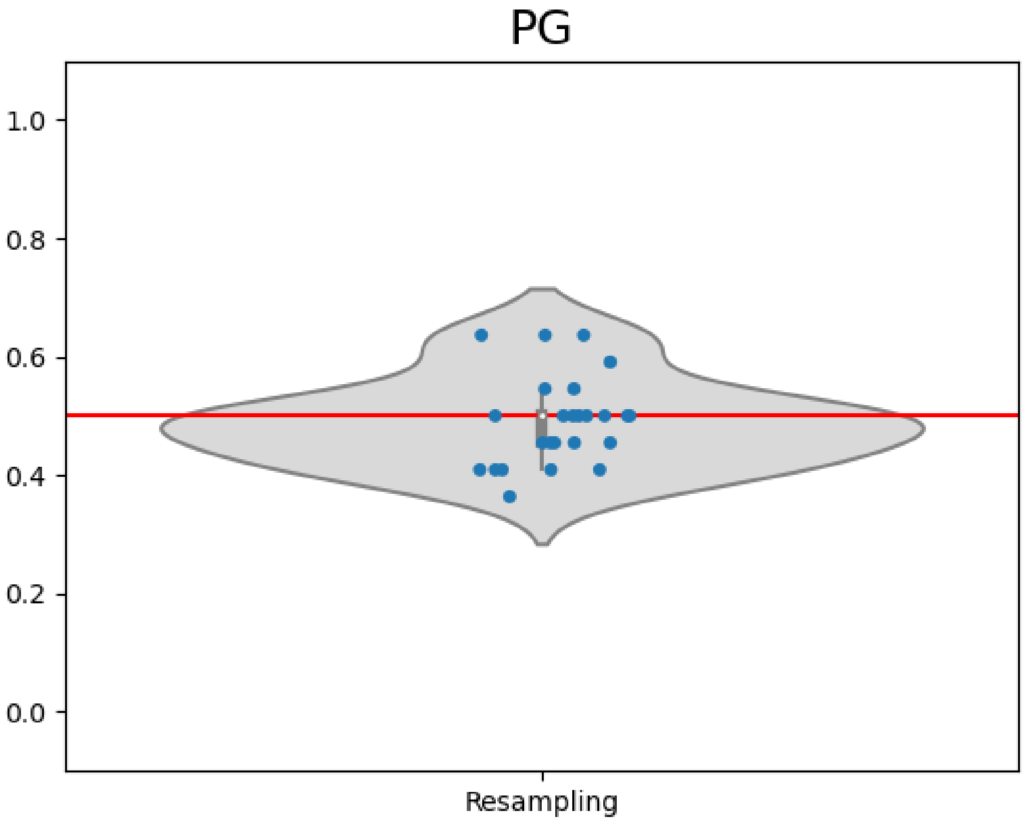
PG
1.0
0.8
0.6
0.4
0.2
0.0
Resampling

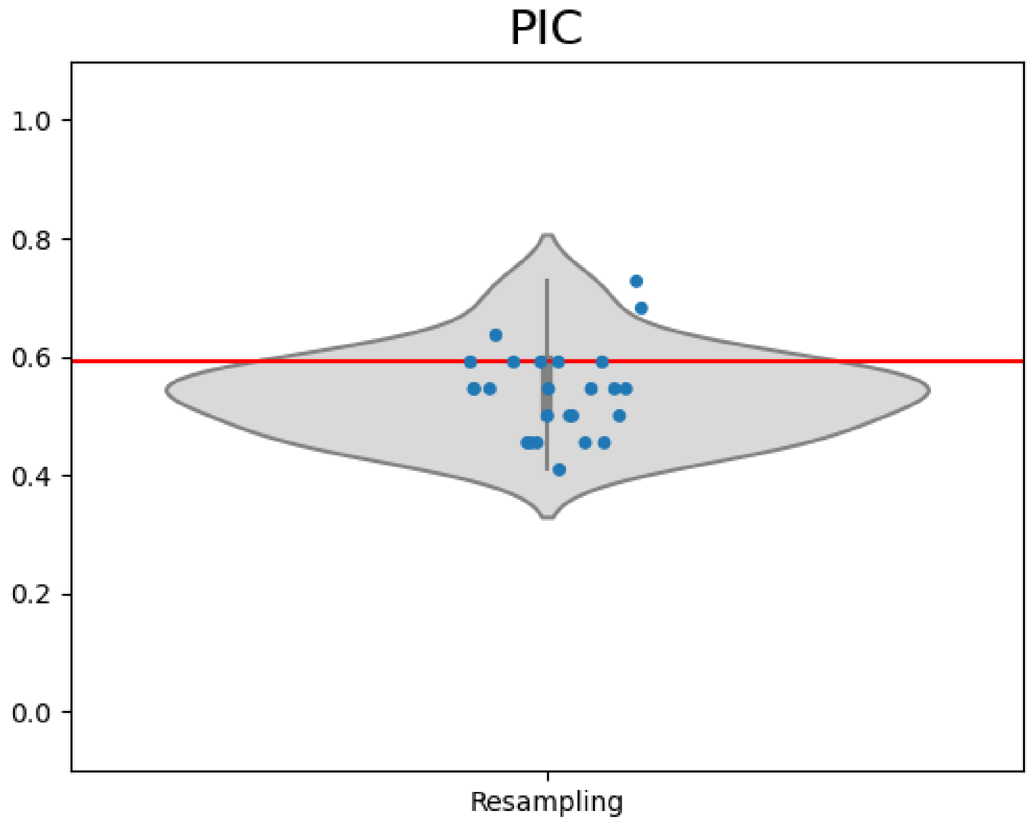
PIC
1.0
0.8
0.6
0.4
0.2
0.0
Resampling

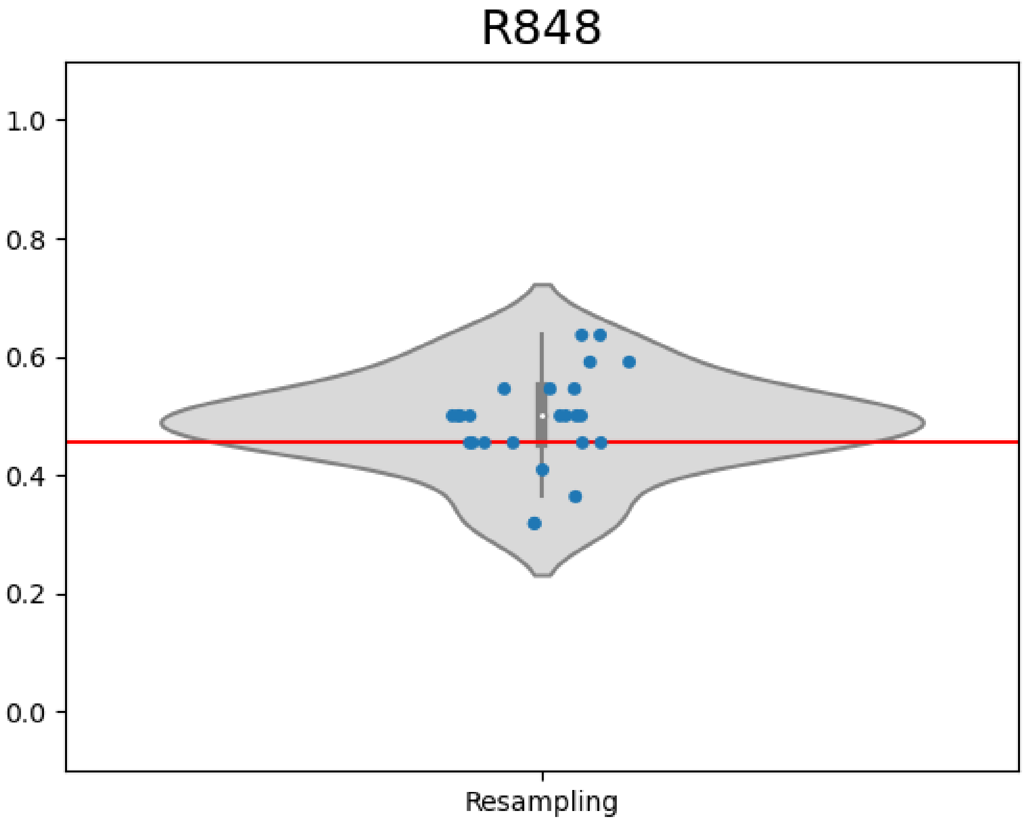
R848
1.0
0.8
0.6
0.4
0.2
0.0
Resampling

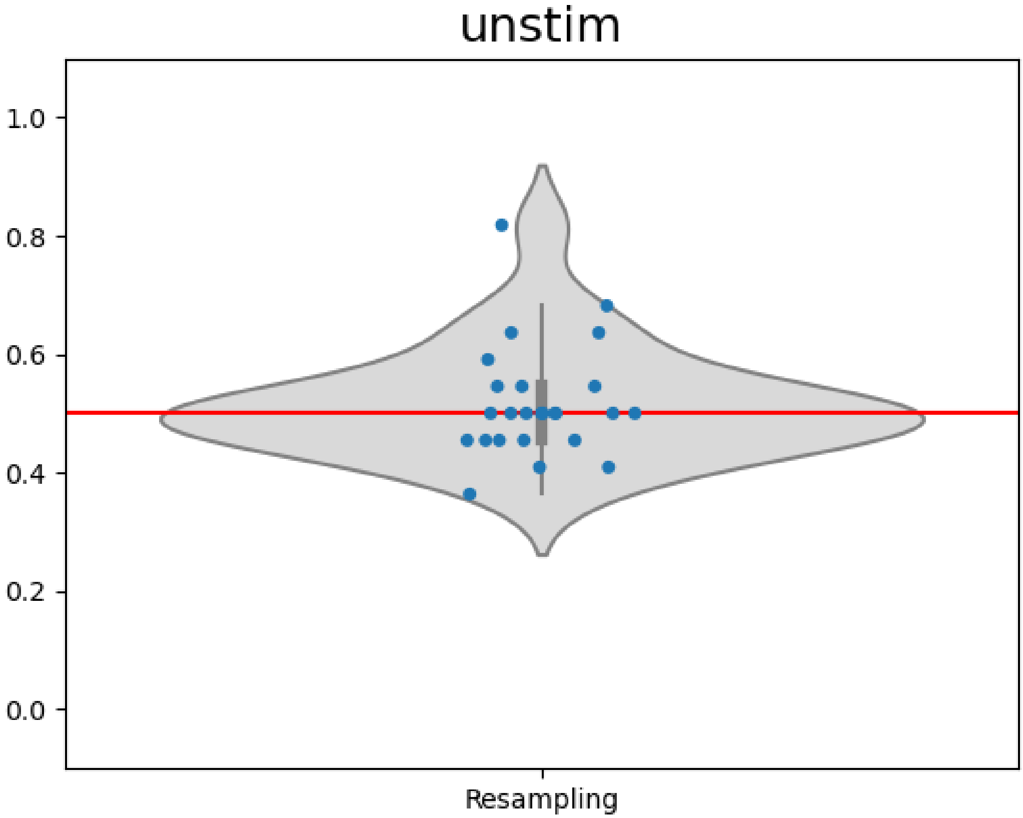
unstim
1.0
0.8
0.6
0.4
0.2
0.0
Resampling
